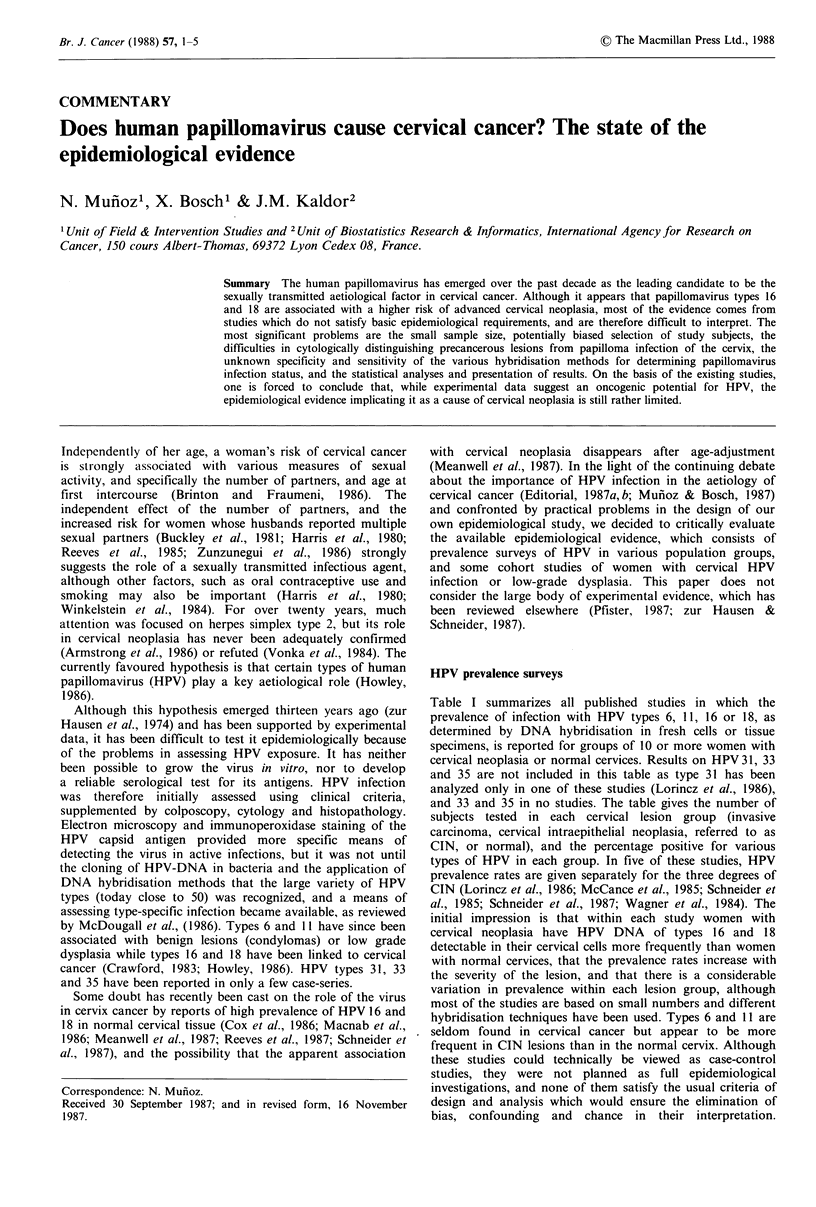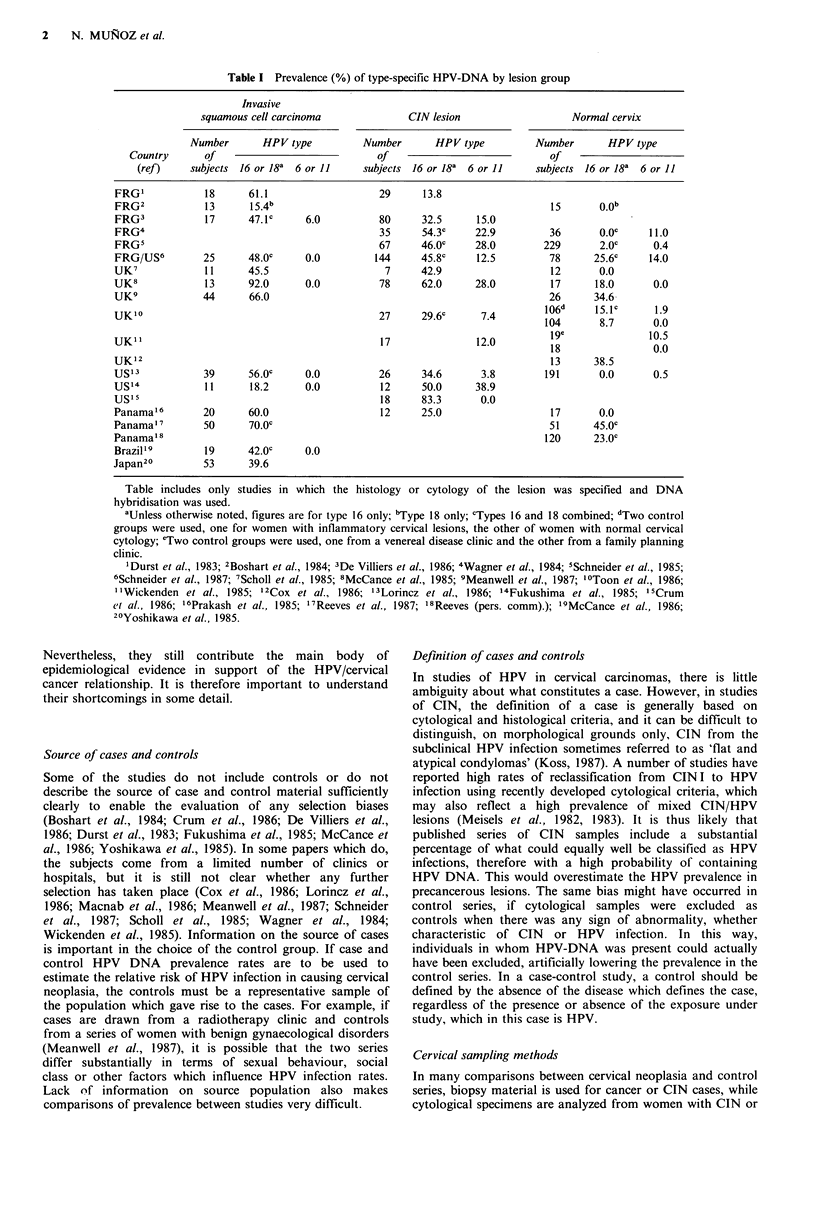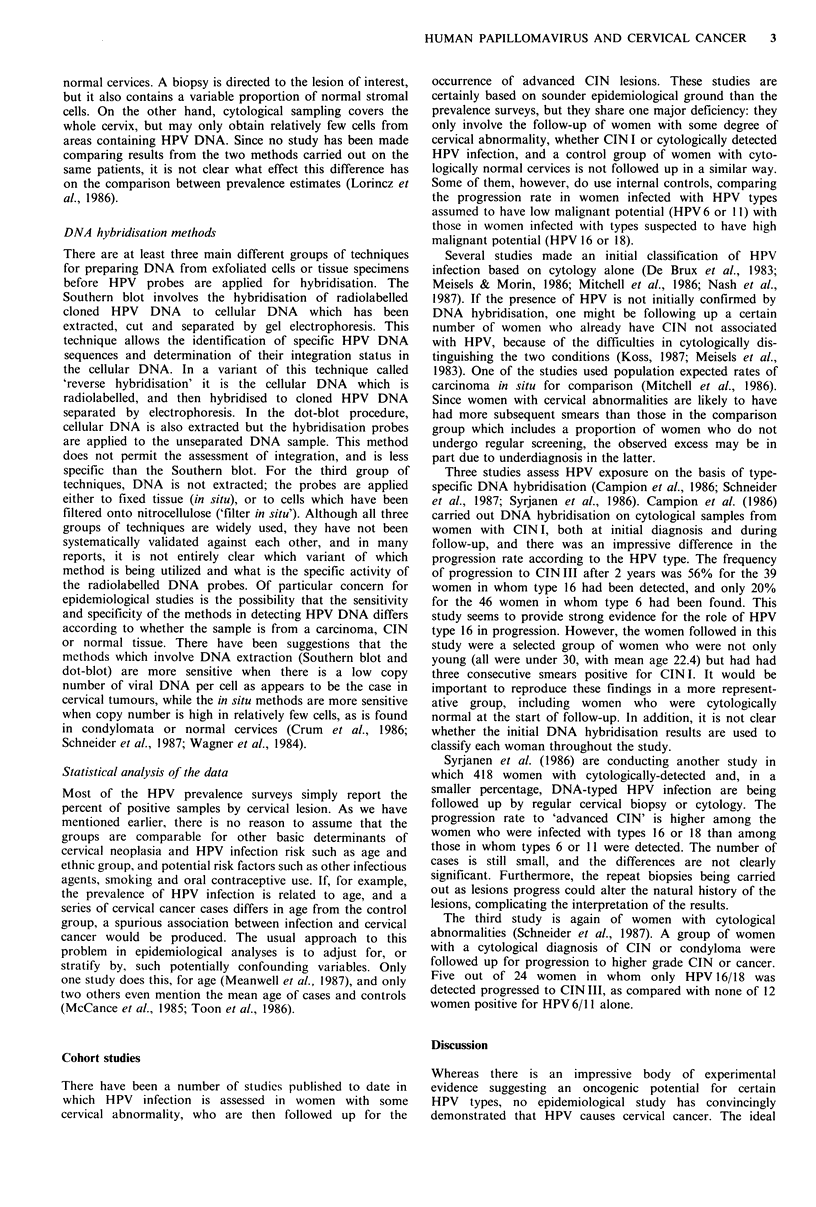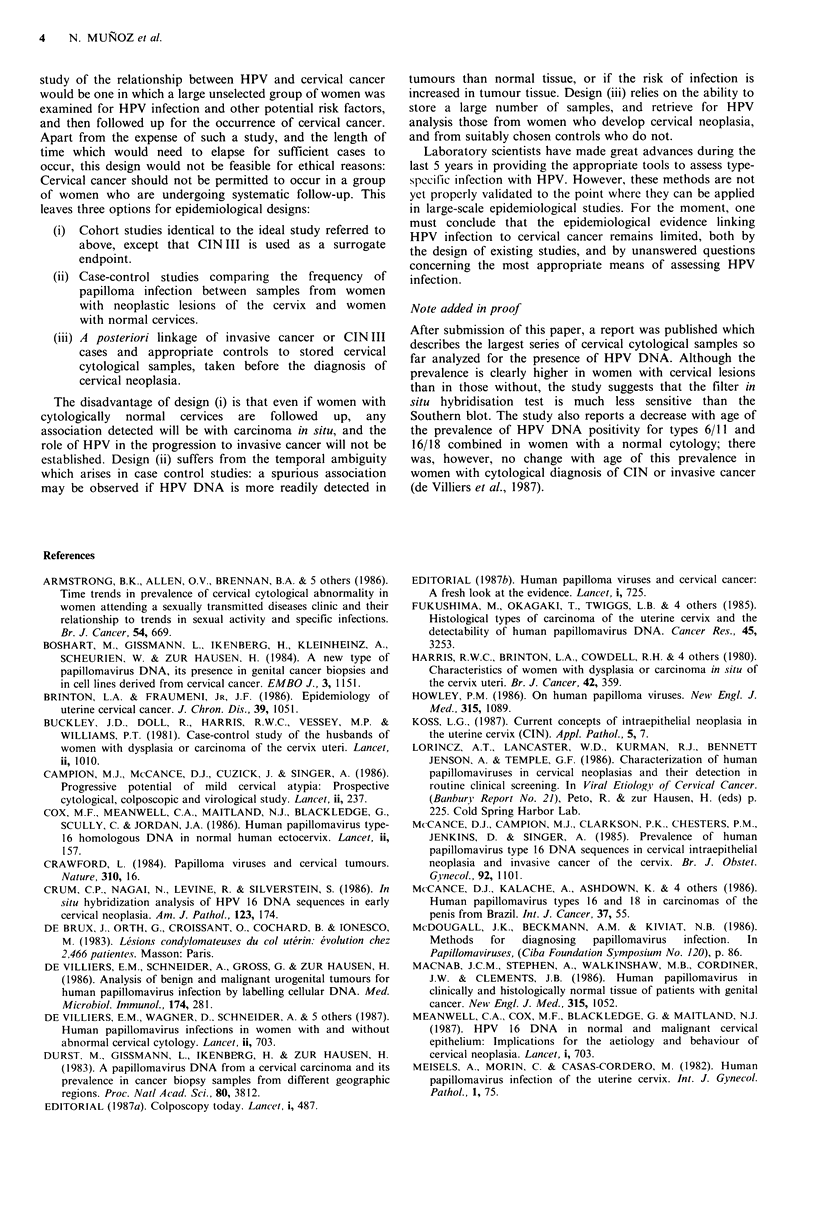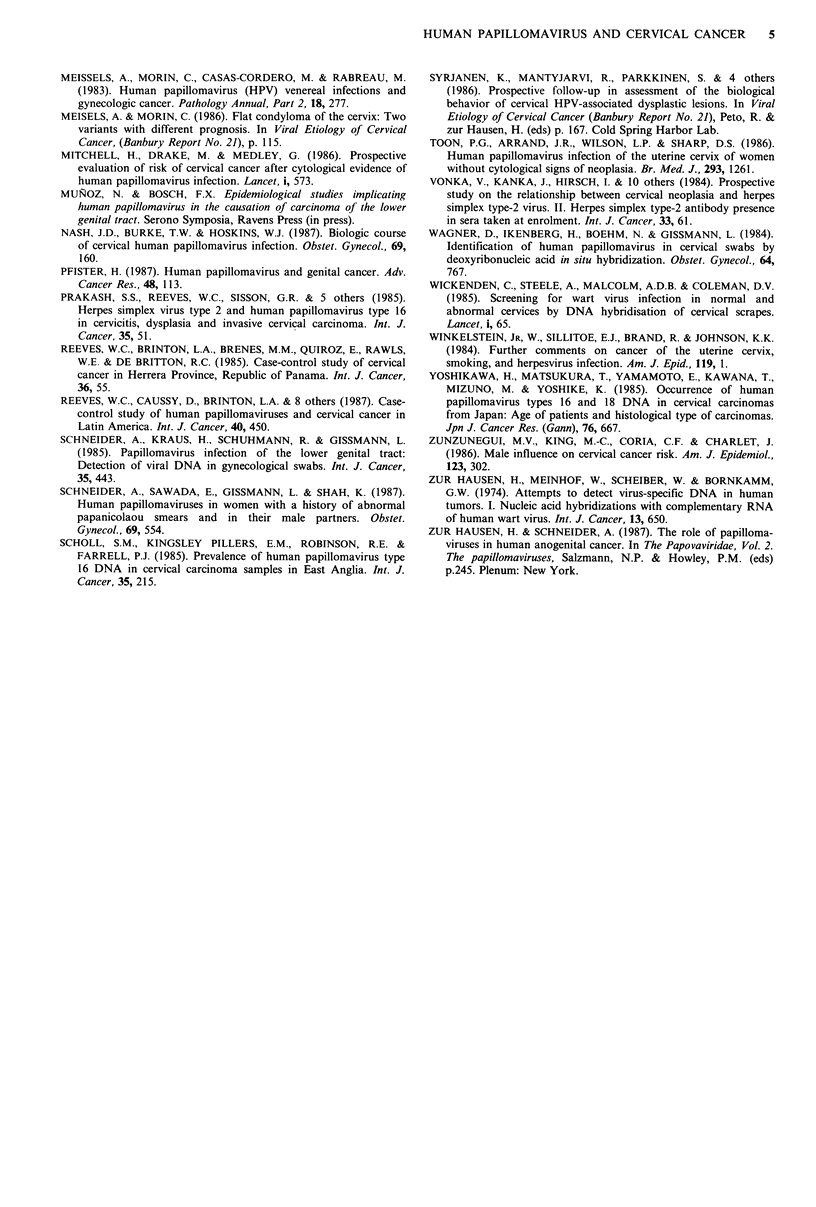# Does human papillomavirus cause cervical cancer? The state of the epidemiological evidence.

**DOI:** 10.1038/bjc.1988.1

**Published:** 1988-01

**Authors:** N. Muñoz, X. Bosch, J. M. Kaldor

**Affiliations:** Unit of Field & Intervention Studies, International Agency for Research on Cancer, Lyon, France.

## Abstract

The human papillomavirus has emerged over the past decade as the leading candidate to be the sexually transmitted aetiological factor in cervical cancer. Although it appears that papillomavirus types 16 and 18 are associated with a higher risk of advanced cervical neoplasia, most of the evidence comes from studies which do not satisfy basic epidemiological requirements, and are therefore difficult to interpret. The most significant problems are the small sample size, potentially biased selection of study subjects, the difficulties in cytologically distinguishing precancerous lesions from papilloma infection of the cervix, the unknown specificity and sensitivity of the various hybridisation methods for determining papillomavirus infection status, and the statistical analyses and presentation of results. On the basis of the existing studies, one is forced to conclude that, while experimental data suggest an oncogenic potential for HPV, the epidemiological evidence implicating it as a cause of cervical neoplasia is still rather limited.


Br. J. Cancer (1988) 57, 1-5

© The Macmillan Press Ltd., 1988

COMMENTARY

### Does human papillomavirus cause cervical cancer? The state of the epidemiological evidence

N. Muñoz[1], X. Bosch[1] & J.M. Kaldor[2]

[1]*Unit of Field & Intervention Studies and* [2]*Unit of Biostatistics Research & Informatics, International Agency for Research on Cancer, 150 cours Albert-Thomas, 69372 Lyon Cedex 08, France.*

**Summary** The human papillomavirus has emerged over the past decade as the leading candidate to be the sexually transmitted aetiological factor in cervical cancer. Although it appears that papillomavirus types 16 and 18 are associated with a higher risk of advanced cervical neoplasia, most of the evidence comes from studies which do not satisfy basic epidemiological requirements, and are therefore difficult to interpret. The most significant problems are the small sample size, potentially biased selection of study subjects, the difficulties in cytologically distinguishing precancerous lesions from papilloma infection of the cervix, the unknown specificity and sensitivity of the various hybridisation methods for determining papillomavirus infection status, and the statistical analyses and presentation of results. On the basis of the existing studies, one is forced to conclude that, while experimental data suggest an oncogenic potential for HPV, the epidemiological evidence implicating it as a cause of cervical neoplasia is still rather limited.

Independently of her age, a woman's risk of cervical cancer is strongly associated with various measures of sexual activity, and specifically the number of partners, and age at first intercourse (Brinton and Fraumeni, 1986). The independent effect of the number of partners, and the increased risk for women whose husbands reported multiple sexual partners (Buckley *et al.*, 1981; Harris *et al.*, 1980; Reeves *et al.*, 1985; Zunzunegui *et al.*, 1986) strongly suggests the role of a sexually transmitted infectious agent, although other factors, such as oral contraceptive use and smoking may also be important (Harris *et al.*, 1980; Winkelstein *et al.*, 1984). For over twenty years, much attention was focused on herpes simplex type 2, but its role in cervical neoplasia has never been adequately confirmed (Armstrong *et al.*, 1986) or refuted (Vonka *et al.*, 1984). The currently favoured hypothesis is that certain types of human papillomavirus (HPV) play a key aetiological role (Howley, 1986).

Although this hypothesis emerged thirteen years ago (zur Hausen *et al.*, 1974) and has been supported by experimental data, it has been difficult to test it epidemiologically because of the problems in assessing HPV exposure. It has neither been possible to grow the virus *in vitro*, nor to develop a reliable serological test for its antigens. HPV infection was therefore initially assessed using clinical criteria, supplemented by colposcopy, cytology and histopathology. Electron microscopy and immunoperoxidase staining of the HPV capsid antigen provided more specific means of detecting the virus in active infections, but it was not until the cloning of HPV-DNA in bacteria and the application of DNA hybridisation methods that the large variety of HPV types (today close to 50) was recognized, and a means of assessing type-specific infection became available, as reviewed by McDougall *et al.*, (1986). Types 6 and 11 have since been associated with benign lesions (condylomas) or low grade dysplasia while types 16 and 18 have been linked to cervical cancer (Crawford, 1983; Howley, 1986). HPV types 31, 33 and 35 have been reported in only a few case-series.

Some doubt has recently been cast on the role of the virus in cervix cancer by reports of high prevalence of HPV 16 and 18 in normal cervical tissue (Cox *et al.*, 1986; Macnab *et al.*, 1986; Meanwell *et al.*, 1987; Reeves *et al.*, 1987; Schneider *et al.*, 1987), and the possibility that the apparent association with cervical neoplasia disappears after age-adjustment (Meanwell *et al.*, 1987). In the light of the continuing debate about the importance of HPV infection in the aetiology of cervical cancer (Editorial, 1987*a,b*; Muñoz & Bosch, 1987) and confronted by practical problems in the design of our own epidemiological study, we decided to critically evaluate the available epidemiological evidence, which consists of prevalence surveys of HPV in various population groups, and some cohort studies of women with cervical HPV infection or low-grade dysplasia. This paper does not consider the large body of experimental evidence, which has been reviewed elsewhere (Pfister, 1987; zur Hausen & Schneider, 1987).

Correspondence: N. Muñoz.
Received 30 September 1987; and in revised form, 16 November 1987.

### HPV prevalence surveys

Table I summarizes all published studies in which the prevalence of infection with HPV types 6, 11, 16 or 18, as determined by DNA hybridisation in fresh cells or tissue specimens, is reported for groups of 10 or more women with cervical neoplasia or normal cervices. Results on HPV 31, 33 and 35 are not included in this table as type 31 has been analyzed only in one of these studies (Lorincz *et al.*, 1986), and 33 and 35 in no studies. The table gives the number of subjects tested in each cervical lesion group (invasive carcinoma, cervical intraepithelial neoplasia, referred to as CIN, or normal), and the percentage positive for various types of HPV in each group. In five of these studies, HPV prevalence rates are given separately for the three degrees of CIN (Lorincz *et al.*, 1986; McCance *et al.*, 1985; Schneider *et al.*, 1985; Schneider *et al.*, 1987; Wagner *et al.*, 1984). The initial impression is that within each study women with cervical neoplasia have HPV DNA of types 16 and 18 detectable in their cervical cells more frequently than women with normal cervices, that the prevalence rates increase with the severity of the lesion, and that there is a considerable variation in prevalence within each lesion group, although most of the studies are based on small numbers and different hybridisation techniques have been used. Types 6 and 11 are seldom found in cervical cancer but appear to be more frequent in CIN lesions than in the normal cervix. Although these studies could technically be viewed as case-control studies, they were not planned as full epidemiological investigations, and none of them satisfy the usual criteria of design and analysis which would ensure the elimination of bias, confounding and chance in their interpretation.

**Table I** Prevalence (%) of type-specific HPV-DNA by lesion group

| Country (*ref*) | *Invasive squamous cell carcinoma* | | | *CIN lesion* | | | *Normal cervix* | | |
|---|---|---|---|---|---|---|---|---|---|
| | *Number of subjects* | *HPV type* | | *Number of subjects* | *HPV type* | | *Number of subjects* | *HPV type* | |
| | | *16 or 18*[a] | *6 or 11* | | *16 or 18*[a] | *6 or 11* | | *16 or 18*[a] | *6 or 11* |
| FRG[1] | 18 | 61.1 | | 29 | 13.8 | | | | |
| FRG[2] | 13 | 15.4[b] | | | | | 15 | 0.0[b] | |
| FRG[3] | 17 | 47.1[c] | 6.0 | 80 | 32.5 | 15.0 | | | |
| FRG[4] | | | | 35 | 54.3[c] | 22.9 | 36 | 0.0[c] | 11.0 |
| FRG[5] | | | | 67 | 46.0[c] | 28.0 | 229 | 2.0[c] | 0.4 |
| FRG/US[6] | 25 | 48.0[c] | 0.0 | 144 | 45.8[c] | 12.5 | 78 | 25.6[c] | 14.0 |
| UK[7] | 11 | 45.5 | | 7 | 42.9 | | 12 | 0.0 | |
| UK[8] | 13 | 92.0 | 0.0 | 78 | 62.0 | 28.0 | 17 | 18.0 | 0.0 |
| UK[9] | 44 | 66.0 | | | | | 26 | 34.6 | |
| UK[10] | | | | 27 | 29.6[c] | 7.4 | 106[d]<br>104 | 15.1[c]<br>8.7 | 1.9<br>0.0 |
| UK[11] | | | | 17 | | 12.0 | 19[e]<br>18 | | 10.5<br>0.0 |
| UK[12] | | | | | | | 13 | 38.5 | |
| US[13] | 39 | 56.0[c] | 0.0 | 26 | 34.6 | 3.8 | 191 | 0.0 | 0.5 |
| US[14] | 11 | 18.2 | 0.0 | 12 | 50.0 | 38.9 | | | |
| US[15] | | | | 18 | 83.3 | 0.0 | | | |
| Panama[16] | 20 | 60.0 | | 12 | 25.0 | | 17 | 0.0 | |
| Panama[17] | 50 | 70.0[c] | | | | | 51 | 45.0[c] | |
| Panama[18] | | | | | | | 120 | 23.0[c] | |
| Brazil[19] | 19 | 42.0[c] | 0.0 | | | | | | |
| Japan[20] | 53 | 39.6 | | | | | | | |

Table includes only studies in which the histology or cytology of the lesion was specified and DNA hybridisation was used.

[a]Unless otherwise noted, figures are for type 16 only; [b]Type 18 only; [c]Types 16 and 18 combined; [d]Two control groups were used, one for women with inflammatory cervical lesions, the other of women with normal cervical cytology; [e]Two control groups were used, one from a venereal disease clinic and the other from a family planning clinic.

[1]Durst *et al.*, 1983; [2]Boshart *et al.*, 1984; [3]De Villiers *et al.*, 1986; [4]Wagner *et al.*, 1984; [5]Schneider *et al.*, 1985; [6]Schneider *et al.*, 1987; [7]Scholl *et al.*, 1985; [8]McCance *et al.*, 1985; [9]Meanwell *et al.*, 1987; [10]Toon *et al.*, 1986; [11]Wickenden *et al.*, 1985; [12]Cox *et al.*, 1986; [13]Lorincz *et al.*, 1986; [14]Fukushima *et al.*, 1985; [15]Crum *et al.*, 1986; [16]Prakash *et al.*, 1985; [17]Reeves *et al.*, 1987; [18]Reeves (pers. comm).); [19]McCance *et al.*, 1986; [20]Yoshikawa *et al.*, 1985.

Nevertheless, they still contribute the main body of epidemiological evidence in support of the HPV/cervical cancer relationship. It is therefore important to understand their shortcomings in some detail.

*Source of cases and controls*

Some of the studies do not include controls or do not describe the source of case and control material sufficiently clearly to enable the evaluation of any selection biases (Boshart *et al.*, 1984; Crum *et al.*, 1986; De Villiers *et al.*, 1986; Durst *et al.*, 1983; Fukushima *et al.*, 1985; McCance *et al.*, 1986; Yoshikawa *et al.*, 1985). In some papers which do, the subjects come from a limited number of clinics or hospitals, but it is still not clear whether any further selection has taken place (Cox *et al.*, 1986; Lorincz *et al.*, 1986; Macnab *et al.*, 1986; Meanwell *et al.*, 1987; Schneider *et al.*, 1987; Scholl *et al.*, 1985; Wagner *et al.*, 1984; Wickenden *et al.*, 1985). Information on the source of cases is important in the choice of the control group. If case and control HPV DNA prevalence rates are to be used to estimate the relative risk of HPV infection in causing cervical neoplasia, the controls must be a representative sample of the population which gave rise to the cases. For example, if cases are drawn from a radiotherapy clinic and controls from a series of women with benign gynaecological disorders (Meanwell *et al.*, 1987), it is possible that the two series differ substantially in terms of sexual behaviour, social class or other factors which influence HPV infection rates. Lack of information on source population also makes comparisons of prevalence between studies very difficult.

*Definition of cases and controls*

In studies of HPV in cervical carcinomas, there is little ambiguity about what constitutes a case. However, in studies of CIN, the definition of a case is generally based on cytological and histological criteria, and it can be difficult to distinguish, on morphological grounds only, CIN from the subclinical HPV infection sometimes referred to as 'flat and atypical condylomas' (Koss, 1987). A number of studies have reported high rates of reclassification from CIN I to HPV infection using recently developed cytological criteria, which may also reflect a high prevalence of mixed CIN/HPV lesions (Meisels *et al.*, 1982, 1983). It is thus likely that published series of CIN samples include a substantial percentage of what could equally well be classified as HPV infections, therefore with a high probability of containing HPV DNA. This would overestimate the HPV prevalence in precancerous lesions. The same bias might have occurred in control series, if cytological samples were excluded as controls when there was any sign of abnormality, whether characteristic of CIN or HPV infection. In this way, individuals in whom HPV-DNA was present could actually have been excluded, artificially lowering the prevalence in the control series. In a case-control study, a control should be defined by the absence of the disease which defines the case, regardless of the presence or absence of the exposure under study, which in this case is HPV.

*Cervical sampling methods*

In many comparisons between cervical neoplasia and control series, biopsy material is used for cancer or CIN cases, while cytological specimens are analyzed from women with CIN or normal cervices. A biopsy is directed to the lesion of interest, but it also contains a variable proportion of normal stromal cells. On the other hand, cytological sampling covers the whole cervix, but may only obtain relatively few cells from areas containing HPV DNA. Since no study has been made comparing results from the two methods carried out on the same patients, it is not clear what effect this difference has on the comparison between prevalence estimates (Lorincz *et al.*, 1986).

*DNA hybridisation methods*

There are at least three main different groups of techniques for preparing DNA from exfoliated cells or tissue specimens before HPV probes are applied for hybridisation. The Southern blot involves the hybridisation of radiolabelled cloned HPV DNA to cellular DNA which has been extracted, cut and separated by gel electrophoresis. This technique allows the identification of specific HPV DNA sequences and determination of their integration status in the cellular DNA. In a variant of this technique called 'reverse hybridisation' it is the cellular DNA which is radiolabelled, and then hybridised to cloned HPV DNA separated by electrophoresis. In the dot-blot procedure, cellular DNA is also extracted but the hybridisation probes are applied to the unseparated DNA sample. This method does not permit the assessment of integration, and is less specific than the Southern blot. For the third group of techniques, DNA is not extracted; the probes are applied either to fixed tissue (*in situ*), or to cells which have been filtered onto nitrocellulose ('filter *in situ*'). Although all three groups of techniques are widely used, they have not been systematically validated against each other, and in many reports, it is not entirely clear which variant of which method is being utilized and what is the specific activity of the radiolabelled DNA probes. Of particular concern for epidemiological studies is the possibility that the sensitivity and specificity of the methods in detecting HPV DNA differs according to whether the sample is from a carcinoma, CIN or normal tissue. There have been suggestions that the methods which involve DNA extraction (Southern blot and dot-blot) are more sensitive when there is a low copy number of viral DNA per cell as appears to be the case in cervical tumours, while the *in situ* methods are more sensitive when copy number is high in relatively few cells, as is found in condylomata or normal cervices (Crum *et al.*, 1986; Schneider *et al.*, 1987; Wagner *et al.*, 1984).

*Statistical analysis of the data*

Most of the HPV prevalence surveys simply report the percent of positive samples by cervical lesion. As we have mentioned earlier, there is no reason to assume that the groups are comparable for other basic determinants of cervical neoplasia and HPV infection risk such as age and ethnic group, and potential risk factors such as other infectious agents, smoking and oral contraceptive use. If, for example, the prevalence of HPV infection is related to age, and a series of cervical cancer cases differs in age from the control group, a spurious association between infection and cervical cancer would be produced. The usual approach to this problem in epidemiological analyses is to adjust for, or stratify by, such potentially confounding variables. Only one study does this, for age (Meanwell *et al.*, 1987), and only two others even mention the mean age of cases and controls (McCance *et al.*, 1985; Toon *et al.*, 1986).

**Cohort studies**

There have been a number of studies published to date in which HPV infection is assessed in women with some cervical abnormality, who are then followed up for the occurrence of advanced CIN lesions. These studies are certainly based on sounder epidemiological ground than the prevalence surveys, but they share one major deficiency: they only involve the follow-up of women with some degree of cervical abnormality, whether CIN I or cytologically detected HPV infection, and a control group of women with cytologically normal cervices is not followed up in a similar way. Some of them, however, do use internal controls, comparing the progression rate in women infected with HPV types assumed to have low malignant potential (HPV 6 or 11) with those in women infected with types suspected to have high malignant potential (HPV 16 or 18).

Several studies made an initial classification of HPV infection based on cytology alone (De Brux *et al.*, 1983; Meisels & Morin, 1986; Mitchell *et al.*, 1986; Nash *et al.*, 1987). If the presence of HPV is not initially confirmed by DNA hybridisation, one might be following up a certain number of women who already have CIN not associated with HPV, because of the difficulties in cytologically distinguishing the two conditions (Koss, 1987; Meisels *et al.*, 1983). One of the studies used population expected rates of carcinoma *in situ* for comparison (Mitchell *et al.*, 1986). Since women with cervical abnormalities are likely to have had more subsequent smears than those in the comparison group which includes a proportion of women who do not undergo regular screening, the observed excess may be in part due to underdiagnosis in the latter.

Three studies assess HPV exposure on the basis of type-specific DNA hybridisation (Campion *et al.*, 1986; Schneider *et al.*, 1987; Syrjanen *et al.*, 1986). Campion *et al.* (1986) carried out DNA hybridisation on cytological samples from women with CIN I, both at initial diagnosis and during follow-up, and there was an impressive difference in the progression rate according to the HPV type. The frequency of progression to CIN III after 2 years was 56% for the 39 women in whom type 16 had been detected, and only 20% for the 46 women in whom type 6 had been found. This study seems to provide strong evidence for the role of HPV type 16 in progression. However, the women followed in this study were a selected group of women who were not only young (all were under 30, with mean age 22.4) but had had three consecutive smears positive for CIN I. It would be important to reproduce these findings in a more representative group, including women who were cytologically normal at the start of follow-up. In addition, it is not clear whether the initial DNA hybridisation results are used to classify each woman throughout the study.

Syrjanen *et al.* (1986) are conducting another study in which 418 women with cytologically-detected and, in a smaller percentage, DNA-typed HPV infection are being followed up by regular cervical biopsy or cytology. The progression rate to 'advanced CIN' is higher among the women who were infected with types 16 or 18 than among those in whom types 6 or 11 were detected. The number of cases is still small, and the differences are not clearly significant. Furthermore, the repeat biopsies being carried out as lesions progress could alter the natural history of the lesions, complicating the interpretation of the results.

The third study is again of women with cytological abnormalities (Schneider *et al.*, 1987). A group of women with a cytological diagnosis of CIN or condyloma were followed up for progression to higher grade CIN or cancer. Five out of 24 women in whom only HPV 16/18 was detected progressed to CIN III, as compared with none of 12 women positive for HPV 6/11 alone.

**Discussion**

Whereas there is an impressive body of experimental evidence suggesting an oncogenic potential for certain HPV types, no epidemiological study has convincingly demonstrated that HPV causes cervical cancer. The ideal study of the relationship between HPV and cervical cancer would be one in which a large unselected group of women was examined for HPV infection and other potential risk factors, and then followed up for the occurrence of cervical cancer. Apart from the expense of such a study, and the length of time which would need to elapse for sufficient cases to occur, this design would not be feasible for ethical reasons: Cervical cancer should not be permitted to occur in a group of women who are undergoing systematic follow-up. This leaves three options for epidemiological designs:

(i) Cohort studies identical to the ideal study referred to above, except that CIN III is used as a surrogate endpoint.

(ii) Case-control studies comparing the frequency of papilloma infection between samples from women with neoplastic lesions of the cervix and women with normal cervices.

(iii) *A posteriori* linkage of invasive cancer or CIN III cases and appropriate controls to stored cervical cytological samples, taken before the diagnosis of cervical neoplasia.

The disadvantage of design (i) is that even if women with cytologically normal cervices are followed up, any association detected will be with carcinoma *in situ*, and the role of HPV in the progression to invasive cancer will not be established. Design (ii) suffers from the temporal ambiguity which arises in case control studies: a spurious association may be observed if HPV DNA is more readily detected in tumours than normal tissue, or if the risk of infection is increased in tumour tissue. Design (iii) relies on the ability to store a large number of samples, and retrieve for HPV analysis those from women who develop cervical neoplasia, and from suitably chosen controls who do not.

Laboratory scientists have made great advances during the last 5 years in providing the appropriate tools to assess type-specific infection with HPV. However, these methods are not yet properly validated to the point where they can be applied in large-scale epidemiological studies. For the moment, one must conclude that the epidemiological evidence linking HPV infection to cervical cancer remains limited, both by the design of existing studies, and by unanswered questions concerning the most appropriate means of assessing HPV infection.

### *Note added in proof*

After submission of this paper, a report was published which describes the largest series of cervical cytological samples so far analyzed for the presence of HPV DNA. Although the prevalence is clearly higher in women with cervical lesions than in those without, the study suggests that the filter *in situ* hybridisation test is much less sensitive than the Southern blot. The study also reports a decrease with age of the prevalence of HPV DNA positivity for types 6/11 and 16/18 combined in women with a normal cytology; there was, however, no change with age of this prevalence in women with cytological diagnosis of CIN or invasive cancer (de Villiers *et al.*, 1987).

### References

ARMSTRONG, B.K., ALLEN, O.V., BRENNAN, B.A. & 5 others (1986). Time trends in prevalence of cervical cytological abnormality in women attending a sexually transmitted diseases clinic and their relationship to trends in sexual activity and specific infections. *Br. J. Cancer*, **54**, 669.

BOSHART, M., GISSMANN, L., IKENBERG, H., KLEINHEINZ, A., SCHEURIEN, W. & ZUR HAUSEN, H. (1984). A new type of papillomavirus DNA, its presence in genital cancer biopsies and in cell lines derived from cervical cancer. *EMBO J.*, **3**, 1151.

BRINTON, L.A. & FRAUMENI, JR, J.F. (1986). Epidemiology of uterine cervical cancer. *J. Chron. Dis.*, **39**, 1051.

BUCKLEY, J.D., DOLL, R., HARRIS, R.W.C., VESSEY, M.P. & WILLIAMS, P.T. (1981). Case-control study of the husbands of women with dysplasia or carcinoma of the cervix uteri. *Lancet*, **ii**, 1010.

CAMPION, M.J., MCCANCE, D.J., CUZICK, J. & SINGER, A. (1986). Progressive potential of mild cervical atypia: Prospective cytological, colposcopic and virological study. *Lancet*, **ii**, 237.

COX, M.F., MEANWELL, C.A., MAITLAND, N.J., BLACKLEDGE, G., SCULLY, C. & JORDAN, J.A. (1986). Human papillomavirus type-16 homologous DNA in normal human ectocervix. *Lancet*, **ii**, 157.

CRAWFORD, L. (1984). Papilloma viruses and cervical tumours. *Nature*, **310**, 16.

CRUM, C.P., NAGAI, N., LEVINE, R. & SILVERSTEIN, S. (1986). *In situ* hybridization analysis of HPV 16 DNA sequences in early cervical neoplasia. *Am. J. Pathol.*, **123**, 174.

DE BRUX, J., ORTH, G., CROISSANT, O., COCHARD, B. & IONESCO, M. (1983). *Lésions condylomateuses du col utérin: évolution chez 2,466 patientes*. Masson: Paris.

DE VILLIERS, E.M., SCHNEIDER, A., GROSS, G. & ZUR HAUSEN, H. (1986). Analysis of benign and malignant urogenital tumours for human papillomavirus infection by labelling cellular DNA. *Med. Microbiol. Immunol.*, **174**, 281.

DE VILLIERS, E.M., WAGNER, D., SCHNEIDER, A. & 5 others (1987). Human papillomavirus infections in women with and without abnormal cervical cytology. *Lancet*, **ii**, 703.

DURST, M., GISSMANN, L., IKENBERG, H. & ZUR HAUSEN, H. (1983). A papillomavirus DNA from a cervical carcinoma and its prevalence in cancer biopsy samples from different geographic regions. *Proc. Natl Acad. Sci.*, **80**, 3812.

EDITORIAL (1987*a*). Colposcopy today. *Lancet*, **i**, 487.

EDITORIAL (1987*b*). Human papilloma viruses and cervical cancer: A fresh look at the evidence. *Lancet*, **i**, 725.

FUKUSHIMA, M., OKAGAKI, T., TWIGGS, L.B. & 4 others (1985). Histological types of carcinoma of the uterine cervix and the detectability of human papillomavirus DNA. *Cancer Res.*, **45**, 3253.

HARRIS, R.W.C., BRINTON, L.A., COWDELL, R.H. & 4 others (1980). Characteristics of women with dysplasia or carcinoma *in situ* of the cervix uteri. *Br. J. Cancer*, **42**, 359.

HOWLEY, P.M. (1986). On human papilloma viruses. *New Engl. J. Med.*, **315**, 1089.

KOSS, L.G., (1987). Current concepts of intraepithelial neoplasia in the uterine cervix (CIN). *Appl. Pathol.*, **5**, 7.

LORINCZ, A.T., LANCASTER, W.D., KURMAN, R.J., BENNETT JENSON, A. & TEMPLE, G.F. (1986). Characterization of human papillomaviruses in cervical neoplasias and their detection in routine clinical screening. In *Viral Etiology of Cervical Cancer*. (*Banbury Report No. 21*), Peto, R. & zur Hausen, H. (eds) p. 225. Cold Spring Harbor Lab.

MCCANCE, D.J., CAMPION, M.J., CLARKSON, P.K., CHESTERS, P.M., JENKINS, D. & SINGER, A. (1985). Prevalence of human papillomavirus type 16 DNA sequences in cervical intraepithelial neoplasia and invasive cancer of the cervix. *Br. J. Obstet. Gynecol.*, **92**, 1101.

MCCANCE, D.J., KALACHE, A., ASHDOWN, K. & 4 others (1986). Human papillomavirus types 16 and 18 in carcinomas of the penis from Brazil. *Int. J. Cancer*, **37**, 55.

MCDOUGALL, J.K., BECKMANN, A.M. & KIVIAT, N.B. (1986). Methods for diagnosing papillomavirus infection. In *Papillomaviruses*, (*Ciba Foundation Symposium No. 120*), p. 86.

MACNAB, J.C.M., STEPHEN, A., WALKINSHAW, M.B., CORDINER, J.W. & CLEMENTS, J.B. (1986). Human papillomavirus in clinically and histologically normal tissue of patients with genital cancer. *New Engl. J. Med.*, **315**, 1052.

MEANWELL, C.A., COX, M.F., BLACKLEDGE, G. & MAITLAND, N.J. (1987). HPV 16 DNA in normal and malignant cervical epithelium: Implications for the aetiology and behaviour of cervical neoplasia. *Lancet*, **i**, 703.

MEISELS, A., MORIN, C. & CASAS-CORDERO, M. (1982). Human papillomavirus infection of the uterine cervix. *Int. J. Gynecol. Pathol.*, **1**, 75.

MEISSELS, A., MORIN, C., CASAS-CORDERO, M. & RABREAU, M. (1983). Human papillomavirus (HPV) venereal infections and gynecologic cancer. *Pathology Annual, Part 2,* **18,** 277.

MEISELS, A. & MORIN, C. (1986). Flat condyloma of the cervix: Two variants with different prognosis. In *Viral Etiology of Cervical Cancer, (Banbury Report No. 21)*, p. 115.

MITCHELL, H., DRAKE, M. & MEDLEY, G. (1986). Prospective evaluation of risk of cervical cancer after cytological evidence of human papillomavirus infection. *Lancet,* **i,** 573.

MUÑOZ, N. & BOSCH, F.X. *Epidemiological studies implicating human papillomavirus in the causation of carcinoma of the lower genital tract.* Serono Symposia, Ravens Press (in press).

NASH, J.D., BURKE, T.W. & HOSKINS, W.J. (1987). Biologic course of cervical human papillomavirus infection. *Obstet. Gynecol.,* **69,** 160.

PFISTER, H. (1987). Human papillomavirus and genital cancer. *Adv. Cancer Res.,* **48,** 113.

PRAKASH, S.S., REEVES, W.C., SISSON, G.R. & 5 others (1985). Herpes simplex virus type 2 and human papillomavirus type 16 in cervicitis, dysplasia and invasive cervical carcinoma. *Int. J. Cancer,* **35,** 51.

REEVES, W.C., BRINTON, L.A., BRENES, M.M., QUIROZ, E., RAWLS, W.E. & DE BRITTON, R.C. (1985). Case-control study of cervical cancer in Herrera Province, Republic of Panama. *Int. J. Cancer,* **36,** 55.

REEVES, W.C., CAUSSY, D., BRINTON, L.A. & 8 others (1987). Case-control study of human papillomaviruses and cervical cancer in Latin America. *Int. J. Cancer,* **40,** 450.

SCHNEIDER, A., KRAUS, H., SCHUHMANN, R. & GISSMANN, L. (1985). Papillomavirus infection of the lower genital tract: Detection of viral DNA in gynecological swabs. *Int. J. Cancer,* **35,** 443.

SCHNEIDER, A., SAWADA, E., GISSMANN, L. & SHAH, K. (1987). Human papillomaviruses in women with a history of abnormal papanicolaou smears and in their male partners. *Obstet. Gynecol.,* **69,** 554.

SCHOLL, S.M., KINGSLEY PILLERS, E.M., ROBINSON, R.E. & FARRELL, P.J. (1985). Prevalence of human papillomavirus type 16 DNA in cervical carcinoma samples in East Anglia. *Int. J. Cancer,* **35,** 215.

SYRJANEN, K., MANTYJARVI, R., PARKKINEN, S. & 4 others (1986). Prospective follow-up in assessment of the biological behavior of cervical HPV-associated dysplastic lesions. In *Viral Etiology of Cervical Cancer (Banbury Report No. 21)*, Peto, R. & zur Hausen, H. (eds) p. 167. Cold Spring Harbor Lab.

TOON, P.G., ARRAND, J.R., WILSON, L.P. & SHARP, D.S. (1986). Human papillomavirus infection of the uterine cervix of women without cytological signs of neoplasia. *Br. Med. J.,* **293,** 1261.

VONKA, V., KANKA, J., HIRSCH, I. & 10 others (1984). Prospective study on the relationship between cervical neoplasia and herpes simplex type-2 virus. II. Herpes simplex type-2 antibody presence in sera taken at enrolment. *Int. J. Cancer,* **33,** 61.

WAGNER, D., IKENBERG, H., BOEHM, N. & GISSMANN, L. (1984). Identification of human papillomavirus in cervical swabs by deoxyribonucleic acid *in situ* hybridization. *Obstet. Gynecol.,* **64,** 767.

WICKENDEN, C., STEELE, A., MALCOLM, A.D.B. & COLEMAN, D.V. (1985). Screening for wart virus infection in normal and abnormal cervices by DNA hybridisation of cervical scrapes. *Lancet,* **i,** 65.

WINKELSTEIN, JR, W., SILLITOE, E.J., BRAND, R. & JOHNSON, K.K. (1984). Further comments on cancer of the uterine cervix, smoking, and herpesvirus infection. *Am. J. Epid.,* **119,** 1.

YOSHIKAWA, H., MATSUKURA, T., YAMAMOTO, E., KAWANA, T., MIZUNO, M. & YOSHIKE, K. (1985). Occurrence of human papillomavirus types 16 and 18 DNA in cervical carcinomas from Japan: Age of patients and histological type of carcinomas. *Jpn J. Cancer Res. (Gann),* **76,** 667.

ZUNZUNEGUI, M.V., KING, M.-C., CORIA, C.F. & CHARLET, J. (1986). Male influence on cervical cancer risk. *Am. J. Epidemiol.,* **123,** 302.

ZUR HAUSEN, H., MEINHOF, W., SCHEIBER, W. & BORNKAMM, G.W. (1974). Attempts to detect virus-specific DNA in human tumors. I. Nucleic acid hybridizations with complementary RNA of human wart virus. *Int. J. Cancer,* **13,** 650.

ZUR HAUSEN, H. & SCHNEIDER, A. (1987). The role of papillomaviruses in human anogenital cancer. In *The Papovaviridae, Vol. 2. The papillomaviruses,* Salzmann, N.P. & Howley, P.M. (eds) p.245. Plenum: New York.